# Tuberculosis Skin Test Screening in the National Tuberculosis Program of Trinidad and Tobago

**DOI:** 10.3390/healthcare8030236

**Published:** 2020-07-27

**Authors:** Vijay Kumar Chattu, Sateesh Sakhamuri, Shastri Motilal, Liam J. Pounder, Vasishma Kanita Persad, Neelmani Pierre, Shivannie Persad, Nikesha Pooran, Akua Mosi Pottinger

**Affiliations:** 1 Department of Medicine, Faculty of Medicine, University of Toronto, Toronto, ON M5S 1A8, Canada; 2 Occupational Medicine Clinic, St. Michael’s Hospital, Toronto, ON M5C 2C5, Canada; 3 Institute of International Relations, The University of the West Indies, St. Augustine, Trinidad and Tobago; 4 Department of Clinical Medical Sciences, Faculty of Medical Sciences, The University of the West Indies, St Augustine, Trinidad and Tobago; ljj.185@gmail.com (L.J.P.); vasishmapersad@gmail.com (V.K.P.); neelmani.pierre@gmail.com (N.P.); shivanniepersad@gmail.com (S.P.); nikesha.pooran@gmail.com (N.P.); mosipottinger@gmail.com (A.M.P.); 5 Eric Williams Medical Sciences Complex, North Central Regional Health Authority, Mount Hope, Trinidad and Tobago; 6 Department of Paraclinical Sciences, Faculty of Medical Sciences, The University of the West Indies, St. Augustine, Trinidad and Tobago; Shastri.Motilal@sta.uwi.edu

**Keywords:** *Mycobacterium tuberculosis*, World Health Organization (WHO), latent TB infection (LTB), tuberculin skin test (TST), Bacillus Calmette–Guerin (BCG), interferon-Gamma releasing assays (IGRAs), Trinidad and Tobago

## Abstract

Globally, a quarter of the population is infected with tuberculosis (TB), caused by *Mycobacterium tuberculosis*. About 5–10% of latent TB infections (LTBI) progress to active disease during the lifetime. Prevention of TB and treating LTBI is a critical component of the World Health Organization’s (WHO) End TB Strategy. This study aims to examine the screening practices for prevention and treatment employed by the National Tuberculosis Program of Trinidad and Tobago in comparison to the WHO’s standard guidelines. A cross-sectional retrospective study was conducted from the TB registers (2018–2019) for persons aged 18 years and above with recorded tuberculin skin test reactions (TST). Bivariate comparisons for categorical variables were made using Chi-square or Fisher’s exact test. Binary logistic regression was used for exploring predictors of TST positivity with adjustment for demographic confounders in multivariable models. Of the total 1972 eligible entries studied, 384 (19.4%) individuals were tested positive with TST. TB contact screening (aOR 2.49; 95% CI 1.65, 3.75) and Bacillus Calmette–Guerin (BCG) vaccination status (aOR 1.66; 95% CI, 1.24 to 2.22) were associated with a positive TST reaction, whereas, preplacement screening failed to show such association when compared to those screened as suspect cases. The findings suggest that TB contact screening and positive BCG vaccination status are associated with TST positivity independent of age and gender.

## 1. Introduction

Tuberculosis (TB) is a highly infectious disease caused by the bacterium *Mycobacterium tuberculosis*. As stated by the World Health Organization (WHO), it is the single leading cause of infection and ranks among the top 10 mortality rates worldwide. In 2018, 10 million persons acquired TB, with more than one million people dying every year due to TB, and between 2000–2007, approximately 54 million lives were saved, bringing the TB incidence to 2% per year globally [1]. Trinidad and Tobago is a high-income southern Caribbean country, has been listed as a low TB burden country by WHO based on its incidence rate of 21 cases per 100,000 [2]. TB exists in two forms, active disease (primary and secondary) and latent infection. Latent TB infection (LTBI) is a dormant state of the bacteria, exhibits immune response to mycobacterial antigens with no symptoms related to disease [3]. About a quarter of the world’s population is estimated to be TB infected [4], with approximately 5–10% likely to develop active TB disease within their lifespan [5]. Despite its significance, no published data is available on latent TB infection in Trinidad and Tobago. Interferon-gamma release assay (IGRA) and tuberculin skin test (TST) are useful to detect LTBI [6]. HIV infected individuals, patients on immunosuppressive therapy, and persons with end-stage renal disease are more susceptible to TB disease [6]. Positive tests for LTBI can also be used to support the diagnosis of active TB, especially in resource-limited settings. However, negative tests cannot rule out active disease in suspect cases as many of them (25%) can exhibit false-negative reactions due to poor nutrition and general health, overwhelming acute illness or weakened immune system [7,8]. Treatment is required for LTBI before its progression into TB disease. The WHO’s End TB Strategy [1], along with the Center for Disease Control and Prevention (CDC), has devised a thorough scheme to prevent and treat all facets of TB.

Prevention of TB and treating LTBI is a critical component of the WHO’s initiative to End TB. In 2018, the UN high-level meeting on TB set a target to reach at least 30 million people with TB preventive treatment for a latent TB infection in the five years 2018–2022. Thus, quality control of TB screening plays a significant role in the National Tuberculosis Program (NTP)’s performance, which caters services to the public through chest clinics by administering TST. Hence, this research was undertaken to (1) examine the factors associated with positive TST and (2) to evaluate the gaps in the current latent TB screening practices of the NTP of Trinidad and Tobago and to compare it to the regional and international standards.

## 2. Experimental Section

### 2.1. Setting 

The NTP piloted by the Ministry of Health in Trinidad and Tobago oversees tuberculosis monitoring within the sister islands [9]. Services offered in this program include diagnostic services (TST, sputum acid-fast bacilli (AFB) microscopy, sputum Xpert MTB-Rif test, chest X-ray, and CT scans) and preventive services (TB contact screening, LTBI management, health education, and research) [2]. IGRA assays have not been introduced yet under the NTP. 

### 2.2. Study Design

A cross-sectional study was conducted at all the chest clinics in the country under the purview of NTP. After obtaining the ethical approval from the relevant authorities, records were collected from the tuberculin skin test (TST) registers, for a total of 10 months between 2018 and 2019. This study was approved by the Campus Research Ethics Committee (CEC) at the University of the West Indies, St. Augustine (Ref: CEC797/11/18). Ethical clearance for data collection from chest clinics was also obtained from the North Central Regional Health Authority (NCRHA) and the Thoracic Medical Director, Caura Hospital.

### 2.3. Participants

All persons aged 18 years and over presenting for screening during the chosen period were included and the data were collected on demographics, symptoms, reaction to TST, TB screening type, and self-reported health conditions.

### 2.4. Data Collection

All the registers were manually reviewed by two researchers independently. Entries for the chosen months were transcribed into data collection sheets, with each entry being assigned a numeric code to ensure confidentiality. The method of administration and interpretation of reaction to TST used by the NTP are as per the CDC guidelines [10].

### 2.5. Administration of Tuberculin Skin Test (TST)

A tuberculin syringe was used, with the needle bevel up, to inject 0.1 mL of purified protein derivative (5 tuberculin units) intra-dermally on the forearm’s inner surface, producing a wheal 6 to 10 mm in diameter. The test reaction is read 48–72 hours later by measuring the size (widest horizontal diameter) of the induration in millimeters (mm) produced.

### 2.6. Interpretation of TST Reaction

Interpretation is based on the size of the induration in association with the persons’ risk of TB infection and progression to disease if infected. An induration of 5 mm or more is considered positive in persons with recent close contact with infectious TB patients, and individuals with HIV, or other immune-suppressive conditions. Induration of 10 mm or more is considered positive in recent immigrants from high prevalence countries, persons living or working in high-risk group settings, injection drug users, and children exposed to high-risk adults. Induration of 15 mm or more is considered positive for persons with no known risk of TB [11].

### 2.7. TB Screening Types

Trinidad and Tobago NTP has classified the indications for performing TST as below:

1. TB suspect screening—for persons displaying signs or symptoms indicative of active TB disease.
2. TB contact screening—for persons who have been in contact with a known infectious case of TB.
3. Preplacement (baseline) screening—required for entry to healthcare institutions or immigration purposes, especially for migration to countries like UK, USA, Canada, certain European countries, Australia, and New Zealand [9].
4. Periodic/annual screening—for healthcare personnel at high risk of exposure to TB.

Individuals with symptoms, risk factors, and radiographic findings suggestive of TB, have been further evaluated with bacteriological tests to rule out active TB disease.

### 2.8. Data Analysis

Descriptive data were presented using frequencies and percentages. Bivariate comparisons for categorical variables were made using Chi-square or Fisher’s exact test. Binary logistic regression was used for exploring predictors of TST positivity with adjustment for demographic confounders in multivariable models. Odds ratios with 95% confidence limits were calculated with and without adjustment. A *p* value of < 0.05 was used to demonstrate statistical significance. IBM, SPSS Version 23.0 (IBM, Armonk, NY, USA) was used for the analysis.

## 3. Results

### 3.1. Subjects Selection

**Figure 1:**
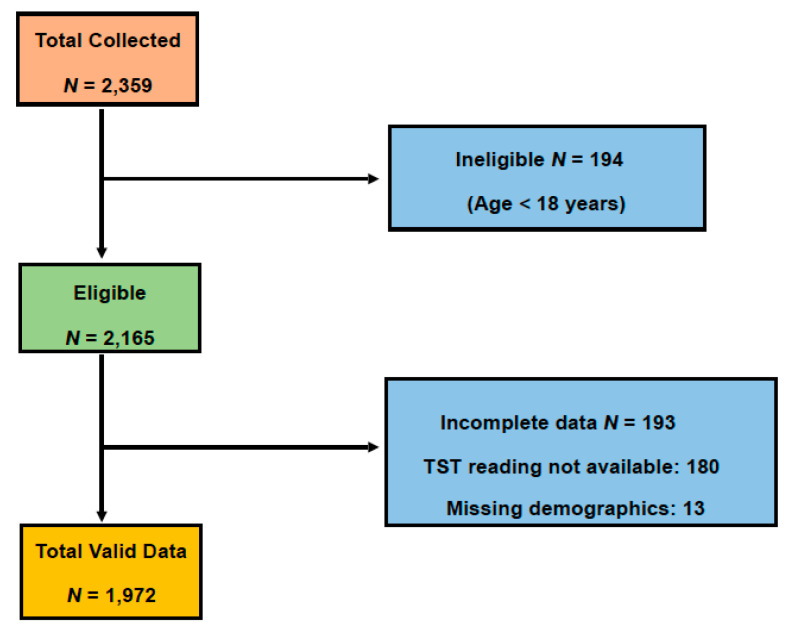
Flowchart showing the process of sample selection.

As seen in Figure 1, over the study duration of ten months (2018–2019), around 2359 tuberculosis screening data entries were collected, and 194 records were excluded as their age was below 18 years. Further, 180 individuals whose TST reaction was not reported, and the other 13 with missing demographics were excluded. The total sample of 1972 records was included in the study.

### 3.2. Demographic, Health, and Screening Details

Of all the participant records studies, most of the individuals screened were between the ages of 18–39 years (48.7%) and females (58.0%). About 20% of the participants reported a cough for more than three weeks. Less than 5% of the individuals reported morbidities such as diabetes, HIV infection, cancer, and history of organ transplantation. About 20% of the studied population reported a history of Bacillus Calmette–Guerin (BCG) vaccination during childhood (Table 1). All these individuals were older than 40 years, as the BCG vaccination practice was discontinued locally in 1976.

Notably, a majority (45.1%) of the TB screenings’ indication has not been reported in the TB register. The most common reported screening indications were TB suspect screening (30.2%), and pre-placement screening (15.0%). Of the total 1972 eligible entries studied, 384 (19.4%) individuals were tested positive with TST. Among the indications of screening, contact screening individuals (31.7%) reacted more positive than other indications (*p* < 0.001) (Table 2). Males (23.6%), and age group 40 and above (24.7%) also reacted frequently positive with TST (*p* < 0.001). Among the health variables, individuals with cough >3 weeks (24.7%), history of BCG vaccination (30.0%), negative or unknown HIV status (18.1% and above), and with diabetes (34.0%) exhibited more positive TST reactions than their counterparts (*p* < 0.05 for all).

### 3.3. Univariate and Multivariate Analysis of Tuberculin Skin Test (TST) Positivity

**Figure 2:**
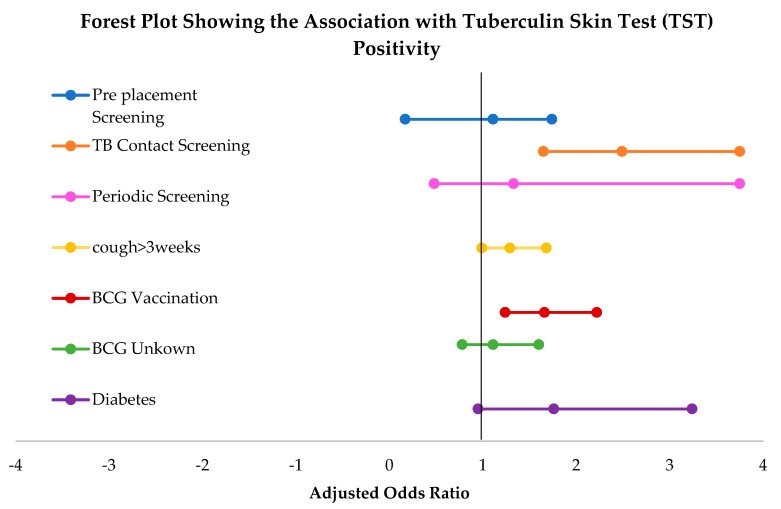
Forest plot showing the association with tuberculin skin test (TST) positivity.

Notably, none of the HIV infected individuals reacted with TST in the study (Table 2). In the univariate analyses (Table 3), comparing with TB suspect case screening, pre-placement screening displayed low odds (0.65) of testing positive with TST. Whereas, TB contacts showed two times increased odds of testing positive. In the multivariate analysis adjusted for age and gender (Table 3), compared to suspect screening, contact screening was associated with more than two times odds of reacting positive with tuberculin test (adjusted OR 2.49; 95% CI 1.65, 3.75), whereas pre-placement and periodic screenings failed to display such association (Figure 2). Among the health variables, BCG vaccination status was associated with increased tuberculin positivity (adjusted OR 1.66; 95% CI 1.24, 2.22) independent of age and gender. In unadjusted analyses, diabetes and cough >3 weeks were also positively correlated with TST positivity. However, such associations were lost after demographic adjustment, as shown in Table 3.

## 4. Discussion

Tuberculosis is a leading cause of global morbidity and mortality. The WHO’s END-TB Strategy was developed to reduce TB incidence and mortality by 90% and 95%, respectively, by 2035 in compared with 2015 [1]. The prevention and management of LTBI is a critical component of achieving this goal [6]. To our knowledge, this is the first study of TB screening practice based on the National Tuberculosis Program in Trinidad and Tobago, and it provides a collated purview of the associations between screening type versus patient demographics, morbidities, and TST interpretations.

We observed from the flow chart (Figure 1) that 9.2% of eligible participants were excluded due to incomplete documentation of patient medical records or persons defaulting from the TST interpretation for a diverse array of reasons. Many motives can be attributed to a person defaulting TB treatment such as ignorance, patient availability to follow-up visits, and low income [12].

Table 1 revealed that 15% of participants were screened as a mandatory entry requirement for their workplace or educational institutes, or immigration purposes (pre-placement screening). Guidelines recommended pre-placement screening for healthcare workers should include a second test (IGRA in addition to TST) based on their risk assessment. Observational studies have shown high case-finding among the screened populations and reduced TB risk in the countries practicing pre-migration TB screening [13,14]. Persons travelling from high TB burden countries constitute one of the fifteen risk groups identified by the WHO as having an increased prevalence of LTBI, increased risk of progression from LTBI to active TB disease, and an increased incidence of active TB [6]. However, screening the migrants from low burden countries like Trinidad and Tobago may not yield much as it correlates with the incidence of TB in the originating country [15].

Only 1.4% of the TSTs were performed for periodic screening of healthcare workers. This practice is consistent with the recent recommendations from the CDC and National Tuberculosis Controllers Association (NTCA), which discouraged routine annual screening for all healthcare workers except for those with increased risk of occupational TB exposure (working with respiratory patients and in emergency departments) [16].

An area for concern regarding screening in the national TB program lies in the number of records from the logbooks observed as unknown screening type (45.1%). Guidelines recommend that only individuals with an appropriate indication, which increases the risk of active TB and who would benefit from treatment from LTBI should be considered for screening [17]. A substantial number of persons (68.4%) registered have reported no history of BCG vaccination. This result was expected considering the demographics observed where the majority of persons screened were found in the 18–39 years age group. Most experts agree that BCG is efficacious against severe forms of childhood TB, but its competency against preventing TB in adults is highly variable. Between 1951 and 1956, the United Nations International Children’s Emergency Fund (UNICEF) and WHO had conducted mass-funded BCG campaigns in the Caribbean [18]. However, these campaigns were not sustained in the years following, as most colonies, Trinidad and Tobago inclusive, struggled to make BCG vaccination an integral part of their TB policy. Globally, 20 (11%) countries abandoned BCG vaccination policy both universally and selectively from their national TB programs [19]. Notably, the majority of these countries are economically possessing upper-middle or high-income statuses. 

Multivariate analyses adjusted for age and gender, as observed in Table 3, showed that the yield with contact screening was highest (two times suspect screening). On the other hand, pre-placement screening and periodic screening failed to exhibit such increased association. BCG vaccination showed an association with TST positivity. It is well known that persons having a history of BCG vaccination after early childhood can reveal false-positive reactions with TST [20]. When considered the pending shortage of TST test materials, this offers a strong case for the use of IGRA’s as complementary to TST. In addition to the lack of BCG sensitization, a single visit for tests indicates IGRA’s preference over TST. Additionally, none of the HIV infected individuals in the study exhibited positive reactions with TST endorsing the addition of IGRAs. It is recognized that persons who are HIV infected may have impaired cell-mediated immunity and fail to react to TST due to anergy [21]. Though similar false-negative reactions can also occur with IGRAs with immunosuppression [22], adding them to TST for individuals with high pre-test probability can improve the precision of TB screening.

Though the guidelines recommended by the international organizations have been followed reasonably by the national TB program, there is a need for increased surveillance and reporting. Instances where screenings for unknown reasons are conducted, as well as the results obtained from pre-placement screening, highlight areas where resources are being wasted. Previous studies estimated that the cost per LTBI screening using TST was USD 22.09, including the reagent and nursing visit costs [23]. Applying this information to our study, within a year, approximately 354 persons would undergo pre-placement screening, roughly computing to USD 7819.86 or TTD 52,862.25 (conversion rate 1 USD = 6.76 TTD). It can be considered wasteful to expend such a large amount on pre-placement screening, which yielded only 9.9% positive TST interpretations, which needs further confirmation with longitudinal studies. Similarly, within the same period, 889 persons have been registered as having been screened for unknown purposes, equating to roughly USD 19,558.00 or TTD 132,212.08, quite a significant figure.

Policymakers should prioritize the indications for targeted LTBI screening based on the observed findings. Contact and healthcare worker periodic screening should be equally prioritized as the suspect TB cases. However, preplacement screening should be rationalized by considering the risk factors. Besides, adding IGRAs to the current TB screening can enhance the national TB program’s competency by improving patient adherence and test efficacy in certain groups. As the CDC projects a scarcity of purified-protein derivative (PPD) tuberculin antigens of tuberculin skin tests, complementing the current screening methods with IGRAs can address such shortages [24].

### Limitations

The study is the first of its kind in the country to examine data on individuals who have been referred for TB screening and factors associated with TST positivity. However, it has some inherent limitations and biases characteristic of any observational and cross-sectional studies with self-reported data. Accuracy of reported symptoms and comorbidities might have been influenced by the participants’ knowledge and subjective perceptions. Moreover, there were around 180 patients whose TST reaction was not reported and another 13 files with missing socio-demographics, which were excluded. Possibility of technical limitations with TST like improper tuberculin handling and interpretation also cannot be ruled out.

## 5. Conclusions

Contact screening has increased the likelihood of positive TST as compared to the suspect case screening. The status of prior BCG vaccination is independently associated with positive TST. There are still a few gaps in the national TB program screening, and inclusion of IGRAs in the screening can offer advantages like avoidance of BCG influence on TB screening and reducing patient visits.

## Figures and Tables

**Table 1:** Frequency of demographics, screening type, and self-reported health variables (*n* = 1972).

Variable	Count
Gender	
Male	829 (42.0%)
Female	1143 (58.0%)
Age group	
18–39 years	961 (48.7%)
40–59 years	620 (31.4%)
60 years and above	391 (19.8%)
Cough >3 weeks	
Yes	401 (20.3%)
No	1571 (79.7%)
BCG Vaccination	
Yes	390 (19.8%)
No	1348 (68.4%)
Unknown	234 (11.9%)
HIV infection	
Yes	20 (1.0%)
No	675 (34.2%)
Unknown	1277 (64.8%)
Diabetes	
Yes	50 (2.5%)
No	1922 (97.5%)
Cancer	
Yes	2 (0.1%)
No	1970 (99.9%)
Organ Transplant	
Yes	4 (0.2%)
No	1968 (99.8%)
Screening Type	
TB suspect screening	596 (30.2%)
Pre-placement screening	295 (15.0%)
Contact screening	164 (8.3%)
Periodic screening	28 (1.4%)
Unknown	889 (45.1%)

**Table 2:** Association of tuberculin skin test (TST) result with other variables (*N* = 1972).

Variable	TST Positive *N* = 384	TST Negative *N* = 1588	* *p* Value
TB Screening Type			
TB suspect screening	111 (18.6%)	485 (81.4%)	0.577
Pre-placement screening	38 (12.9%)	257 (87.1%)	<0.001
TB contact screening	52 (31.7%)	112 (68.3%)	<0.001
Periodic screening	5 (17.9%)	23 (82.1%)	0.99
Unknown	178 (20.0%)	711 (80.0%)	0.607
Gender			
Male	196 (23.6%)	633 (76.4%)	<0.001
Female	188 (16.4%)	955 (83.6%)	
Age group			
18–39 years	134 (13.9%)	827 (86.1%)	<0.001
40–59 years	153 (24.7%)	467 (75.3%)	<0.001
60 years and above	97 (24.8%)	294 (75.2%)	<0.001
Cough >3weeks			
Yes	99 (24.7%)	302 (75.3%)	0.004
No	285 (18.1%)	1286 (81.9%)	
BCG Vaccination			
Yes	117 (30.0%)	273 (70.0%)	<0.001
No	223 (16.5%)	1125 (83.5%)	<0.001
Unknown	44 (18.8%)	190 (81.2%)	0.860
HIV infection			
Yes	0 (0.0%)	20 (100.0%)	0.021
No	122 (18.1%)	553 (81.9%)	0.281
Unknown	262 (20.5%)	1015 (79.5%)	0.122
Diabetes			
Yes	17 (34.0%)	33 (66.0%)	0.017
No	367 (19.1%)	1555 (80.9%)	

*—significant at *p* < 0.05 level.

**Table 3:** Univariate and multivariate analysis of tuberculin skin test (TST) positivity with selected associated factors.

Variables	Category	Univariate Model	^#^ Multivariate Model
		OR 95% CI	OR 95% CI
TB Screening	Pre-placement	0.65 (0.43–0.96)	1.11 (0.71–1.74)
	TB contact	2.03 (1.38–2.99)	2.49 (1.65–3.75)
	Periodic	0.95 (0.35–2.95)	1.33 (0.48–3.75)
	TB suspect *	Ref	Ref
Cough for >3 weeks	Yes	1.48 (1.14–1.92)	1.29 (0.99–1.68)
	No *	Ref	Ref
BCG Vaccination	Yes	2.16 (1.67–2.80)	1.66 (1.24–2.22)
	Unknown	1.17 (0.82–1.67)	1.11 (0.78–1.60)
	No *	Ref	Ref
Diabetes	Yes	2.18 (1.20–3.96)	1.76 (0.95–3.24)
	No *	Ref	Ref

^#^ Adjusted for age and gender; * Reference category.

## References

[1] World Health Organization (2019). Global Tuberculosis Report. https://www.who.int/tb/publications/global_report/en/.

[2] Jagger A., Reiter-Karam S., Hamada Y., Getahun H. (2018). National policies on the management of latent tuberculosis infection: Review of 98 countries. Bull. World Health Organ..

[3] Getahun H., Matteelli A., Abubakar I., Aziz M.A., Baddeley A., Barreira D., Boon S.D., Gutierrez S.M.B., Bruchfeld J., Burhan E. (2015). Management of latent Mycobacterium tuberculosis infection: WHO guidelines for low tuberculosis burden countries. Eur. Respir. J..

[4] Houben R.M.G.J., Dodd P.J. (2016). The Global Burden of Latent Tuberculosis Infection: A Re-estimation Using Mathematical Modelling. PLoS Med..

[5] Comstock G.W., Livesay V.T., Woolpert S.F. (1974). The prognosis of a positive tuberculin reaction in childhood and adolescence. Am. J. Epidemiol..

[6] Latent Tuberculosis Infection: Updated and Consolidated Guidelines for Programmatic Management. https://apps.who.int/iris/bitstream/handle/10665/260233/9789241550239-eng.pdf.

[7] Holden M., Dubin M.R., Diamond P.H. (1971). Frequency of negative intermediate-strength tuberculin sensitivity in patients with active tuberculosis. N. Engl. J. Med..

[8] American Thoracic Society (2000). Diagnostic Standards and Classification of Tuberculosis in Adults and Children. This official statement of the American Thoracic Society and the Centers for Disease Control and Prevention was adopted by the ATS Board of Directors, July 1999. This statement was endorsed by the Council of the Infectious Disease Society of America, September 1999. Am. J. Respir. Crit. Care Med..

[9] The Ministry of Health—Trinidad and Tobago National Tuberculosis control Programme. http://www.health.gov.tt/sitepages/default.aspx?id=284.

[10] Fact Sheets. https://www.cdc.gov/tb/publications/factsheets/testing/skintesting.htm.

[11] Muture B.N., Keraka M.N., Kimuu P.K., Kabiru E.W., Ombeka V.O., Oguya F. (2011). Factors associated with default from treatment among tuberculosis patients in Nairobi province, Kenya: A case control study. BMC Public Health.

[12] Arshad S., Bavan L., Gajari K., Paget S.N.J., Baussano I. (2010). Active screening at entry for tuberculosis among new immigrants: A systematic review and meta-analysis. Eur. Respir. J..

[13] Kranzer K., Afnan-Holmes H., Tomlin K., Golub J.E., Shapiro A.E., Schaap A., Corbett E.L., Lönnroth K., Glynn J.R. (2013). The benefits to communities and individuals of screening for active tuberculosis disease: A systematic review. Int. J. Tuberc. Lung Dis..

[14] Aldridge R.W., Yates T.A., Zenner D., White P.J., Abubakar I., Hayward A. (2014). Pre-entry screening programmes for tuberculosis in migrants to low-incidence countries: A systematic review and meta-analysis. Lancet Infect. Dis..

[15] Sosa L.E., Njie G.J., Lobato M.N., Bamrah S.M., Buchta W., Casey M.L., Goswami N.D., Gruden M., Hurst B.J., Khan A.R. (2019). Tuberculosis Screening, Testing, and Treatment of U.S. Health Care Personnel: Recommendations from the National Tuberculosis Controllers Association and CDC, 2019. Morb. Mortal. Wkly. Rep..

[16] Lewinsohn D.M., Leonard M.K., LoBue P.A., Cohn D.L., Daley C.L., Desmond E., Keane J., Lewinsohn D.A., Loeffler A.M., Mazurek G.H. (2017). Official American Thoracic Society/Infectious Diseases Society of America/Centers for Disease Control and Prevention Clinical Practice Guidelines: Diagnosis of Tuberculosis in Adults and Children. Clin. Infect. Dis..

[17] Zwerling A., Behr M., Verma A., Brewer T., Menzies D., Pai M. (2011). The BCG World Atlas: A Database of Global BCG Vaccination Policies and Practices. PLoS Med..

[18] Altink H. (2014). ‘Fight TB with BCG’: Mass Vaccination Campaigns in the British Caribbean, 1951–6. Med. Hist..

[19] Pooransingh S., Sakhamuri S. (2020). Need for BCG Vaccination to Prevent TB in High-Incidence Countries and Populations. Emerg. Infect. Dis..

[20] Wang L., Turner M.O., Elwood R.K., Schulzer M., FitzGerald J.M. (2002). A meta-analysis of the effect of Bacille Calmette Guérin vaccination on tuberculin skin test measurements. Thorax.

[21] Cobelens F., Egwaga S., Ginkel T., Muwinge H., Matee M., Borgdorff M. (2006). Tuberculin Skin Testing in Patients with HIV Infection: Limited Benefit of Reduced Cutoff Values. Clin. Infect. Dis..

[22] Aabye M.G., Ravn P., PrayGod G., Jeremiah K., Mugomela A., Jepsen M., Faurholt D., Range N., Friis H., Changalucha J. (2009). The impact of HIV infection and CD4 cell count on the performance of an interferon gamma release assay in patients with pulmonary tuberculosis. PLoS ONE.

[23] Linas B.P., Wong A.Y., Freedberg K.A., Horsburgh C.R. (2011). Priorities for screening and treatment of latent tuberculosis infection in the United States. Am. J. Respir. Crit. Care Med..

[24] CDC (2019). Nationwide Shortage of Tuberculin Skin Test Antigens: CDC Recommendations for Patient Care and Public Health Practice. MMWR Morb. Mortal. Wkly. Rep..

